# HIIT Promotes M2 Macrophage Polarization and Sympathetic Nerve Density to Induce Adipose Tissue Browning in T2DM Mice

**DOI:** 10.3390/biom14030246

**Published:** 2024-02-20

**Authors:** Yifan Guo, Qilong Zhang, Dan Yang, Peijie Chen, Weihua Xiao

**Affiliations:** 1 Shanghai Key Lab of Human Performance, Shanghai University of Sport, Shanghai 200438, China; 2111516027@sus.edu.cn (Y.G.); qilongzhang@sus.edu.cn (Q.Z.); 2221516016@sus.edu.cn (D.Y.); 2 The Key Lab of Exercise and Health Sciences of Ministry of Education, Shanghai University of Sport, Shanghai 200438, China

**Keywords:** white adipose tissue browning, high-intensity interval training, macrophage, innervation, sympathetic nervous system, type 2 diabetes mellitus

## Abstract

Browning of white adipose tissue (WAT) is a focus of research in type 2 diabetes mellitus (T2DM) and metabolism, which may be a potential molecular mechanism for high-intensity interval training (HIIT) to improve T2DM. In this study, male *C57BL/6J* wild-type mice were subjected to an 8-week HIIT regimen following T2DM induction through a high-fat diet (HFD) combined with streptozotocin (STZ) injection. We found that HIIT improved glucose metabolism, body weight, and fat mass in T2DM mice. HIIT also decreased adipocyte size and induced browning of WAT. Our data revealed a decrease in *TNFα* and an increase in *IL-10* with HIIT, although the expression of chemokines *MCP-1* and *CXCL14* was increased. We observed increased pan-macrophage infiltration induced by HIIT, along with a simultaneous decrease in the expression of M1 macrophage markers (iNOS and CD11c) and an increase in M2 macrophage markers (Arg1 and CD206), suggesting that HIIT promotes M2 macrophage polarization. Additionally, HIIT upregulated the expression of *Slit3* and neurotrophic factors (*BDNF* and *NGF*). The expression of the sympathetic marker tyrosine hydroxylase (TH) and the nerve growth marker *GAP43* was also increased, demonstrating the promotion of sympathetic nerve growth and density by HIIT. Notably, we observed macrophages co-localizing with TH, and HIIT induced the accumulation of M2 macrophages around sympathetic nerves, suggesting a potential association between M2 macrophages and increased density of sympathetic nerves. In conclusion, HIIT induces adipose tissue browning and improves glucose metabolism in T2DM mice by enhancing M2 macrophage polarization and promoting sympathetic nerve growth and density.

## 1. Introduction

In recent years, the global prevalence of diabetes has reached alarming levels, with approximately 537 million adults (ages 20–79) affected in 2021. Among all cases, type 2 diabetes mellitus (T2DM) accounts for a significant majority, comprising 90–95% of the total [1]. It is widely acknowledged that T2DM is influenced by modifiable lifestyle factors such as poor diet and physical inactivity, which contribute to adipose tissue dysfunction, insulin resistance, and chronic inflammation, ultimately leading to the development and progression of the disease.

Non-pharmacological interventions, including various forms of physical activity, have been recognized as effective therapies for managing T2DM and improving overall health outcomes. These interventions encompass aerobic exercise training, resistance exercise training, combined exercise training, and high-intensity interval training (HIIT) [2]. Among these, HIIT has garnered substantial attention due to its potential to elicit profound physiological and metabolic adaptations in a time-efficient manner. However, the precise mechanisms underlying the beneficial effects of HIIT in T2DM remain elusive.

The emergence of beige adipocytes within white adipose tissue (WAT) has been implicated in multiple aspects of glucose uptake, insulin resistance, and lipid metabolism, all of which play crucial roles in the pathogenesis of T2DM [3,4,5]. The unique characteristics of beige fat, such as the presence of multilocular lipid droplets, a high mitochondrial content, and the expression of uncoupling protein 1 (UCP1), enable it to exhibit brown-like activity, thus leading to its designation as “WAT browning”. This phenomenon has gained considerable attention in diabetes and metabolic research due to its potential to modulate whole-body energy metabolism and its inducibility even in adulthood.

While the involvement of WAT browning in exercise-induced improvements in systemic metabolism has been recognized, the underlying mechanisms remain incompletely understood. Of particular interest in recent years is the interplay between macrophages and sympathetic neurons in WAT browning. Macrophages represent a key component of the inflammatory processes associated with metabolic diseases and can be broadly classified into two distinct polarization states: classically activated macrophages (M1) with a proinflammatory phenotype and alternatively activated macrophages (M2) with an anti-inflammatory phenotype [6,7,8]. It has been observed that M2 macrophages in adipose tissue directly interact with sympathetic nerves to exert beneficial metabolic effects by modulating catecholamine tone [9], rather than through the release of catecholamines themselves [10]. Moreover, M2 macrophages promote the outgrowth of sympathetic neurites, resulting in increased neuronal activity, enhanced local catecholamine synthesis, and subsequent WAT browning [11]. In parallel, sympathetic nerve activity maintains an anti-inflammatory environment within adipose tissue by inhibiting the expression of TNF-α in macrophages [12].

Although exercise has been reported to impact the infiltration and polarization status of adipose tissue macrophages, most studies have focused on aerobic/endurance training in individuals without T2DM or in obese subjects [13]. Consequently, limited data are available regarding the effects of HIIT in individuals with T2DM. Furthermore, the influence of exercise on local sympathetic nerves within adipose tissue remains poorly understood. The aim of this study was to investigate the effects of an 8-week HIIT program on WAT browning in T2DM mice and to elucidate the roles of sympathetic nerves and macrophages in this process.

## 2. Method and Materials

### 2.1. Animals

In this study, 4-week-old male C57BL/6J mice were obtained from the Model Animal Research Center of Nanjing University (Nanjing, China). During the experiments, the mice were individually housed in ventilated cages at a temperature of 23 to 25 °C in the SPF-level animal laboratory of Shanghai University of Sports. To acclimate to the conditions, all mice were fed normal chow (D12450J; 3.85 kcal/g, 10% kcal from fat, 20% kcal from protein, SYSE Ltd., Suzhou, China) for one week prior to the intervention. Food and water were provided ad libitum to the mice. The lighting schedule consisted of 12 h of light and 12 h of darkness, while the humidity was maintained at 50%. The animal study was reviewed and approved by the Ethics Review Committee for Animal Experimentation of Shanghai University of Sport (Approval No. 102772019DW009 and Approval Date 7 March 2019).

The mice were randomly divided into 3 groups (8 mice in each group): control or healthy animal (CON), type 2 diabetes mellitus (T2DM), and T2DM mice subjected to high-intensity interval training (T2DM-EX).

### 2.2. Induction of T2DM

To establish a T2DM animal model, mice were fed a high-fat diet (D12492; 5.24 kcal/g, 60% kcal from fat, 20% kcal from protein, SYSE Ltd., Suzhou, China) for 12 weeks. They were then injected intraperitoneally with STZ (100 mg/kg/body weight) dissolved in citrate buffer (pH = 4.5). Stable blood glucose concentrations were measured 5 days after STZ injection, and a glucometer was used to estimate blood glucose levels. Blood glucose levels above 16.7 mmol/L were considered indicative of confirmed type 2 diabetes mellitus. Mice in the CON group were fed normal chow and received injections of the same dose of citrate buffer. The diets of the mice in each group were maintained throughout the experimental intervention.

### 2.3. High-Intensity Interval Training

Before initiating the training protocol, the animals underwent a 1-week familiarization period with the exercise regimen. Following the familiarization period, HIIT was performed on a running platform inclined at 25°, with 5 sessions per week for a duration of 8 weeks. Each session comprised 10 sets, with each set consisting of 4 min of exercise followed by 2 min of rest. The treadmill speed was gradually increased over time, starting at 16 m/min during the first week. Subsequently, the speed was increased by 2 m/min per week for the initial four weeks, and then by 1 m/min per week for the remaining four weeks, reaching a final speed of 26 m/min.

### 2.4. Glucose Tolerance Test (GTT)

For all mice, the GTT was tested at the end of the 8-week exercise intervention, with fasting started 12 h before the test and water consumed ad libitum. Fasting blood glucose was measured with a glucometer after the tail tips of the mice were amputated, followed by intraperitoneal injection of glucose (1 g/mL) according to the body weight of the mice. Blood glucose levels were measured at 15, 30, 60, 90, and 120 min after glucose injection. The AUC of glucose tolerance was calculated.

### 2.5. Insulin Tolerance Test (ITT)

For ITT, all mice fasted with water supply for 6 h on the day of the experiment. Each mouse received an i.p. injection of 0.75 U/kg insulin after baseline blood glucose was determined. At 15, 30, 60, 90, and 120 min after injection, blood glucose levels were measured. The AUC of insulin tolerance was calculated.

### 2.6. Adiposity Index

After 8 weeks of exercise intervention, mice were sacrificed 36 h following the completion of the last exercise session. The fat depots (inguinal, epididymal, and perirenal) were anatomically dissected, photographed, and weighed. The adiposity index was calculated by summing the weights of the three fat depots and expressing it as a percentage of the body weight.

### 2.7. Hematoxylin and Eosin (HE) Staining

After fixing the inguinal adipose tissue with 4% PFA for 24 h, the tissue was cut into small pieces measuring 1 cm × 1 cm × 1 cm. These pieces were then dehydrated and embedded in paraffin. The wax blocks were further sectioned into 4 μm slices, which were mounted onto slides. The sections were dewaxed and rehydrated using xylene and graded alcohol. In order to observe the morphology and size of the adipocytes, the sections were stained with HE using a standard protocol. An Olympus microscope was used to capture images of six randomly selected fields per section (n = 3 for each group). The area of adipocytes in the HE-stained sections was determined and calculated using ImageJ. At least 100 adipocytes were counted per field.

### 2.8. Immunohistochemistry Staining

For the localization and semi-quantification of UCP1, sections were subjected to pretreatment with 3% H_2_O_2_ and sodium citrate antigen repair solution. Following that, they were blocked with 5% BSA for 1 h and incubated overnight with primary antibodies against UCP1 (1:500, Cell Signaling Technology, Boston, MA, USA, 72298). On the subsequent day, the sections were incubated with a secondary antibody for 1 h at room temperature, followed by staining with streptavidin–biotin complex and DAB. An Olympus microscope was used to capture images of six randomly selected fields per section (n = 3 for each group). Integrated optical density (IOD) values at each field of view were calculated using the IPP 6.0 image analysis software.

### 2.9. Immunofluorescence Staining

The sections were incubated in 5% bovine serum albumin for 2 h, followed by overnight incubation at 4 °C with the following primary antibodies: rat anti-Mac2 antibody (1:500, Cedarlane, Burlington, ON, Canada, CL8942AP), rabbit anti-iNOS antibody (1:500, Abcam, Cambridge, UK, ab15323), rabbit anti-Arg1 antibody (1:500, Cell Signaling Technology, Boston, MA, USA, 93668), and sheep anti-TH antibody (1:500, Novus Biologicals, CO, USA, NB300-110). Next, the sections were incubated with the following secondary antibodies: Alexa 555-Donkey Anti-Rat IgG (1:1000, Abcam, ab150154), Alexa 488-Donkey Anti-Rabbit IgG (1:1000, Abcam, ab150073), and Alexa 647-Donkey Anti-sheep IgG (1:1000, Abcam, ab150179). Finally, the sections were stained with DAPI for 5 min at room temperature to visualize the nuclei. Six randomly selected fields per section (n = 3 for each group) were captured using a Zeiss laser confocal scanning microscope. ImageJ software (version 1.53, National Institutes of Health, Bethesda, USA) was utilized for statistical analysis of the images and to calculate the number of single-label-positive stained cells and the percentage of double- or triple-label-positive stained areas.

### 2.10. RNA Extraction and Gene Expression Analysis

Total RNA was extracted from 100 mg of inguinal adipose tissue using 1 mL of TRIZOL reagent (Thermo Scientific, Dreiech, Germany) following the manufacturer’s instructions. The extracted RNA was quantified using Thermo Scientific NanoDrop™ One microvolume spectrophotometers. Subsequently, cDNA was synthesized using the RevertAid First Strand cDNA Synthesis Kit (Thermo Scientific, Dreiech, Germany) and amplified using the ChamQ Universal SYBR qPCR Master Mix (Vazyme, Nanjing, China, Q711). Quantitative real-time PCR was conducted on a Step One PlusTM PCR system (Thermo Scientific, Dreiech, Germany). The relative expression of the gene of interest was determined using GAPDH as a housekeeping gene through the −ΔΔCT method. The primers utilized are listed in Table 1.

### 2.11. Western Blotting

Standard techniques were used for immunoblotting. Briefly, proteins from inguinal adipose tissue homogenates were separated by SDS-PAGE and transferred onto PVDF membranes. The membranes were blocked with 5% skimmed milk for 1 h at room temperature. Subsequently, they were incubated overnight at 4 °C with primary antibodies against TH (1:250, Cell Signaling Technology, Boston, MA, USA, 58844) and β-actin, followed by a wash and incubation with HRP-conjugated secondary antibodies. After cleaning the membrane again with TBST, it was placed under a developer for enhanced chemiluminescence detection and photography. ImageJ software (National Institutes of Health, Bethesda, MD, USA) was used to calculate the grayscale values of the target proteins.

### 2.12. Statistical Analysis

All data are presented as mean ± SEM. Two-group comparison was analyzed by Student *t*-test. Three-group comparison was analyzed by one-way ANOVA (SPSS version 20.0; SPSS, Chicago, IL, USA) followed by the Games–Howell test. For all tests, a *p* value < 0.05 was considered significant.

## 3. Results

### 3.1. Exercise Intervention Improves Glucose Tolerance and Insulin Sensitivity in T2DM Mice

**Figure 1:**
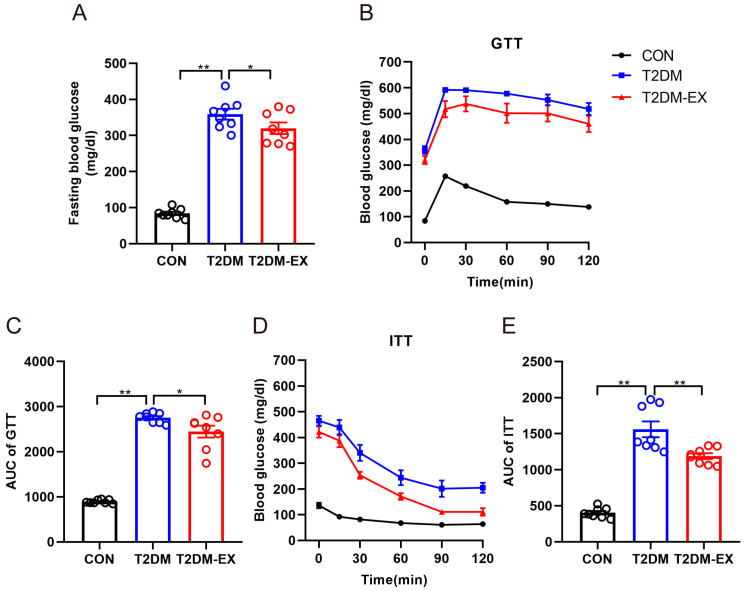
The effects of exercise intervention on glucose metabolism in T2DM mice. (**A**) Fasting blood glucose of the three groups at the end of 8 weeks of exercise. (**B**,**C**) Blood glucose levels and their AUC at 0, 15, 30, 60, 90, and 120 min after glucose injection. (**D**,**E**) Blood glucose levels and their AUC at 0, 15, 30, 60, 90, and 120 min after insulin injection. * *p* < 0.05, ** *p* < 0.01. Data are expressed as mean ± SEM. *n* = 8 per group.

At the end of the 8-week intervention, as expected, the fasting blood glucose of the mice in the T2DM group was much higher than that of the CON mice. When comparing the non-exercised T2DM mice to the exercise intervention group, a significant reduction in fasting glucose was observed in the T2DM mice (Figure 1A). GTT and ITT were utilized to further investigate the impact of the exercise intervention on metabolic function. The results of IP-GTT confirmed that T2DM mice cleared glucose less efficiently than T2DM-EX mice after the intraperitoneal injection of glucose (Figure 1B). Moreover, the area under the curve (AUC) of GTT was significantly lower in the T2DM-EX group compared to the T2DM mice (Figure 1C). The findings from IP-ITT demonstrated that T2DM mice exhibited classic insulin resistance, whereas the exercise intervention partially improved insulin sensitivity in T2DM mice (Figure 1D,E).

### 3.2. Exercise Intervention Reduces Adiposity in T2DM Mice

**Figure 2:**
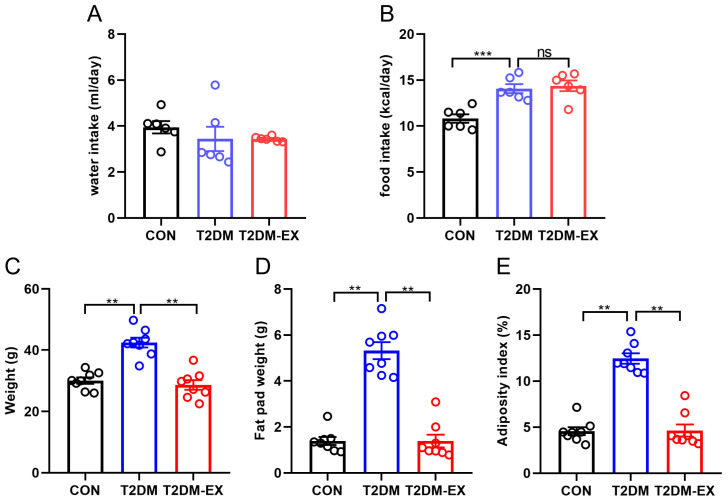
The effects of exercise intervention on body weight and fat weight in T2DM mice. (**A**) Daily water intake in CON and T2DM mice and T2DM-EX mice. (**B**) Daily food intake in CON and T2DM mice and T2DM-EX mice. (**C**) Body weight of CON, T2DM, and T2DM-EX mice after intervention. (**D**,**E**) Total weight of inguinal, epididymal, and perirenal adipose tissue and its ratio to body weight. “ns” indicates no significance, ** *p* < 0.01, *** *p* < 0.001. Data are expressed as mean ± SEM. *n* = 8 per group.

The findings revealed that neither T2DM nor exercise had a significant impact on water intake in mice (Figure 2A). However, there was a notable difference in daily caloric intake between mice fed a high-fat diet and those on a normal diet, with the former consuming significantly more calories (Figure 2B). Interestingly, exercise did not influence food intake. After 8 weeks of HIIT, the mice in the exercise group exhibited significantly lower body weight (Figure 2C). We collected and weighed the inguinal fat, epididymal fat, and perirenal fat and found that the total fat pad weight was significantly higher in the non-exercised T2DM mice compared to the control and exercised groups (Figure 2D). A similar trend was observed when analyzing the adiposity index, which is calculated based on the ratio of fat weight to body weight (Figure 2E).

### 3.3. Exercise Intervention Induces iWAT Browning in T2DM Mice

**Figure 3:**
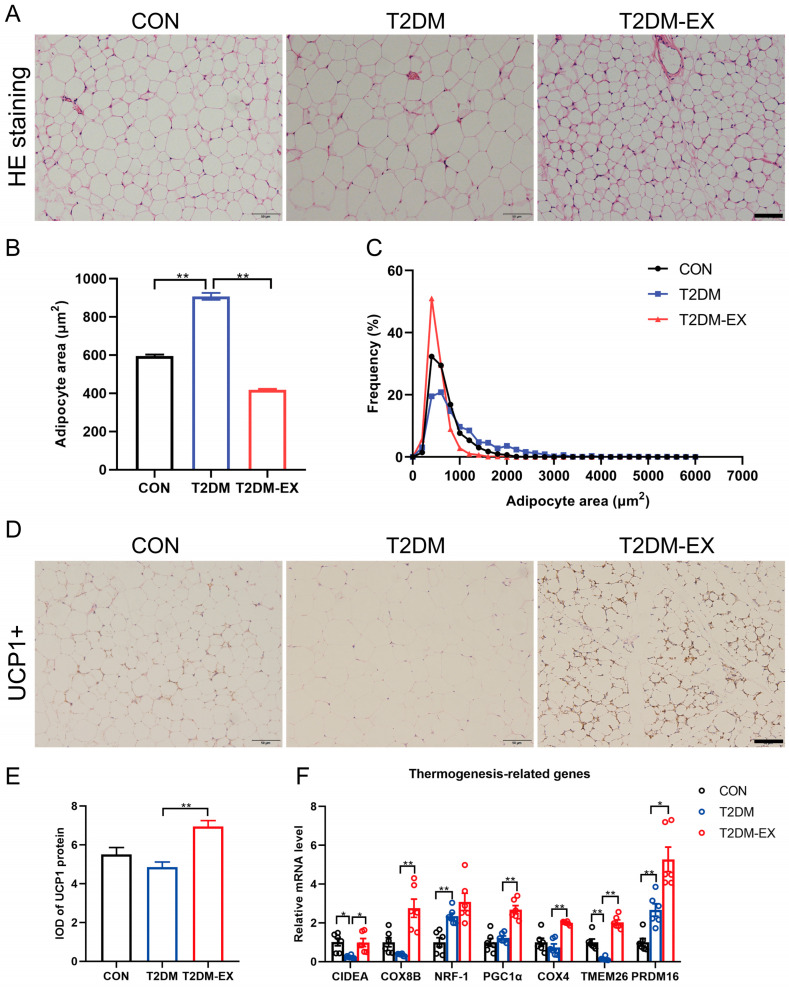
The effects of exercise intervention on browning-related markers. (**A**) Representative images of iWAT with hematoxylin and eosin staining of histological sections from control, T2DM, and T2DM-EX mice. (**B**,**C**) Analysis of adipocyte size and frequency of distribution in HE-stained sections by ImageJ. Three samples per group, 6 fields per section, minimum 80 cells counted per image. (**D**,**E**) Representative images of UCP1 immunohistochemical staining of iWAT in control, T2DM, and T2DM-EX mice and their integrated optical density (IOD) value statistics. Three samples per group, 6 fields per section. (**F**) Thermogenesis gene expression in iWAT, *n* = 6 per group. * *p* < 0.05, ** *p* < 0.01. Scale bar = 50 μm; data are expressed as mean ± SEM.

By performing HE staining of inguinal white adipose tissue (iWAT) and quantifying the cross-sectional area of the cells, we observed that the area of subcutaneous adipocytes was significantly larger in T2DM mice compared to the control group (Figure 3A). The increase in mean adipocyte size was primarily due to adipocyte hypertrophy, as indicated by the increased number of larger adipocytes observed in the frequency distribution of adipocyte sizes. Furthermore, exercise intervention led to a significant reduction in fat cell size (Figure 3B,C). Concurrently, UCP1 staining revealed distinctive brown-like characteristics in the iWAT of T2DM-EX mice (Figure 3D,E). Consistently, exercise significantly upregulated the mRNA levels of thermogenesis-related genes, including CIDEA, COX8B, PGC1α, COX4, TMEM26, and PRDM16 (Figure 3F). These findings suggest that an 8-week exercise intervention induces browning of iWAT in T2DM mice.

### 3.4. The Effects of Exercise Intervention and T2DM on Inflammatory Factors in iWAT

**Figure 4:**
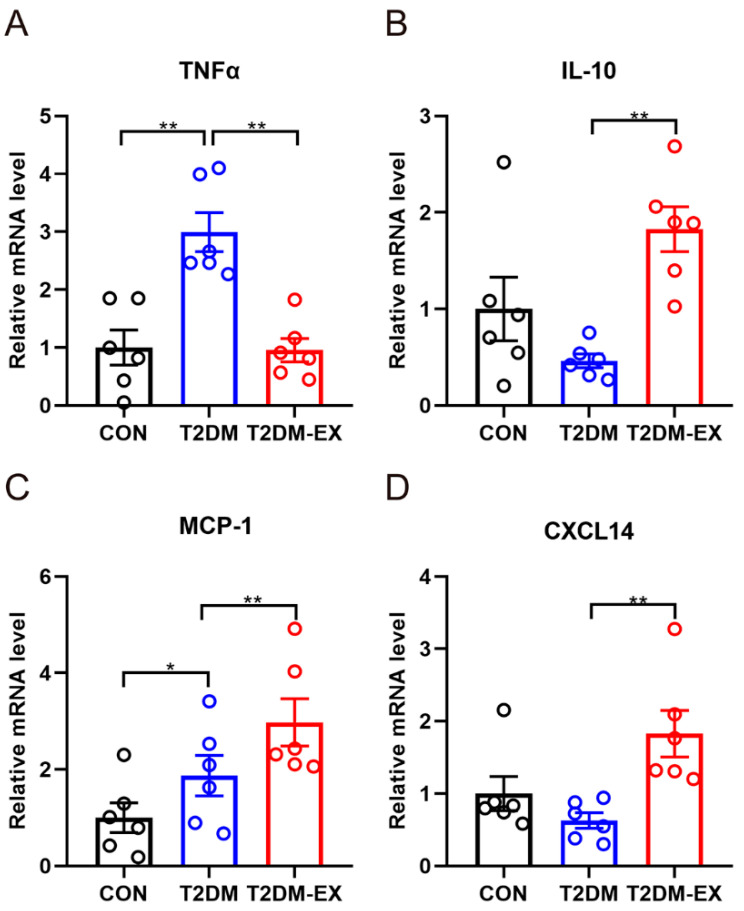
The effects of exercise and T2DM on inflammatory factors in iWAT. (**A**–**D**) The mRNA expression levels of TNFα, IL-10, MCP-1, and CXCL14, *n* = 6. * *p* < 0.05, ** *p* < 0.01. Data are expressed as mean ± SEM.

To investigate the inflammatory status of subcutaneous adipose tissue samples, we analyzed the gene expression of TNF-α and IL-10 as key inflammatory markers. We observed a significant increase in the expression of the proinflammatory marker TNF-α in T2DM mice, while exercise intervention resulted in a decrease in TNF-α expression (Figure 4A). Conversely, the anti-inflammatory marker IL-10 showed the opposite trend, with a significant decrease in IL-10 expression in the T2DM group, and exercise significantly increased IL-10 mRNA levels (Figure 4B). Additionally, we examined the expression of MCP-1, a key chemokine involved in monocyte/macrophage migration and infiltration. The results demonstrated that T2DM led to a significant increase in MCP-1 compared to the CON group, and interestingly, exercise further elevated MCP-1 mRNA levels (Figure 4C). Furthermore, we observed that exercise induced a significant increase in CXCL14 (Figure 4D), a chemokine known to recruit M2 macrophages [14].

### 3.5. The Effects of Exercise Intervention on Macrophage Infiltration and Polarization in T2DM Mice

**Figure 5:**
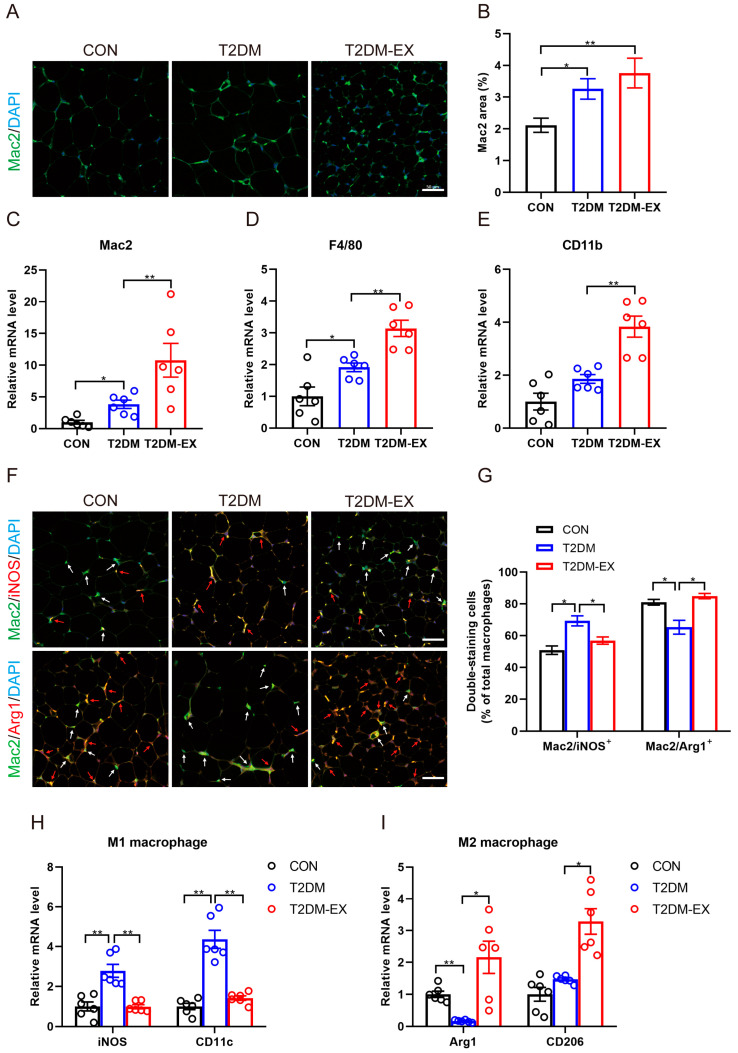
The effects of exercise intervention on macrophage infiltration and polarization in T2DM mice. (**A**) Representative images of Mac2 immunofluorescence staining of iWAT in control, T2DM, and T2DM-EX mice, scale bar = 50 μm. (**B**) Percentage of Mac2-positive area in total image, 3 samples per group, 6 fields per section. (**C**–**E**) The mRNA expression levels of pan-macrophage markers (Mac2, F4/80, and CD11b). (**F**) Representative images showing the double-positive staining of Mac2 and iNOS, Mac2 and Arg1. Scale bar = 50 μm. White arrows indicate Mac2 single-positive cells; red arrows indicate the double-positive cells. Green: Mac2; red: iNOS or Arg1; blue: nucleus stained by DAPI. (**G**) The ratio of double-positive cells to Mac2 single-positive cells. M1 represents Mac2^+^iNOS^+^ and M2 represents Mac2^+^Arg1^+^. Three samples per group, 6 fields per section. (**H**) The mRNA levels of M1-type macrophage markers (iNOS, CD11c) in iWAT. (**I**) The mRNA levels of M2-type macrophage markers (CD206, Arg1) in iWAT. * *p* < 0.05, ** *p* < 0.01. Data are expressed as mean ± SEM.

Initially, we hypothesized that HIIT might improve the inflammatory state by reducing macrophage infiltration, similar to what has been observed with chronic moderate-intensity aerobic exercise [15]. However, the increased expression of chemokines suggested that HIIT may actually promote macrophage infiltration. To investigate this hypothesis, we analyzed the expression of pan-macrophage markers (Mac2, F4/80, and CD11b) in iWAT. Compared to control mice, the percentage of Mac2-positive cells was significantly higher in the iWAT of T2DM mice, indicating macrophage infiltration (Figure 5A,B). Interestingly, the percentage of Mac2-positive cells and the mRNA levels of pan-macrophage markers were further increased in the exercise group mice (Figure 5A–E). These data suggest that HIIT promotes macrophage infiltration in iWAT.

Given the increased macrophage infiltration in iWAT induced by HIIT, but with an improved inflammatory status, we further propose that it may promote a phenotype switch of macrophages from the M1 to the M2 state. Immunofluorescence staining demonstrated a significant increase in M1 macrophages (Mac2^+^iNOS^+^ cells) in the iWAT of non-exercising T2DM mice, whereas M2 macrophages (Mac2^+^Arg1^+^ cells) predominated in the exercise group (Figure 5F,G). Consistent with these findings, HIIT attenuated the increase in iNOS and CD11c gene expression induced by T2DM, while significantly upregulating the mRNA levels of Arg1 and CD206 (Figure 5H,I). The results above demonstrate that 8 weeks of HIIT training induced M2 macrophage polarization accompanied by increased macrophage infiltration.

### 3.6. Exercise Intervention Increases Sympathetic Nerve Density and Partially Activates Its Activity in iWAT

**Figure 6:**
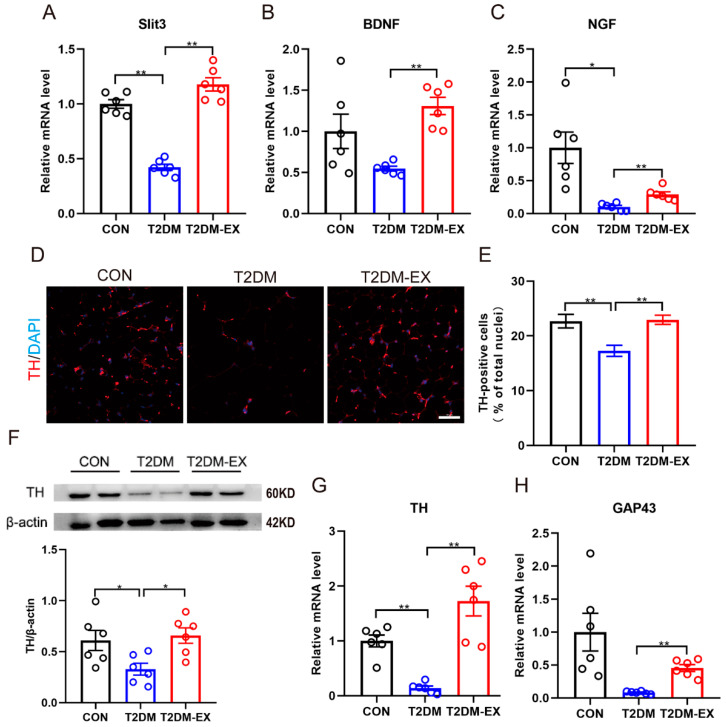
The effects of exercise and T2DM on local sympathetic nerves in iWAT. (**A**–**C**) The mRNA expression levels of Slit3, BDNF, and NGF. (**D**) Representative images of TH immunofluorescence staining of iWAT in control, T2DM, and T2DM-EX mice, scale bar = 50 μm. (**E**) Ratio of the number of TH-positive cells to the number of DAPI-stained nuclei. Three samples per group, 6 fields per section. (**F**) TH protein expression was analyzed by Western blot and quantified using ImageJ in iWAT collected from CON, T2DM, and T2DM-EX group mice. n = 6 per group. (**G**,**H**) The mRNA expression levels of TH and GAP43, *n* = 6 per group. * *p* < 0.05, ** *p* < 0.01. Data are expressed as mean ± SEM. Original images of (**F**) can be found in Supplementary Materials.

It has been reported that cold-induced secretion of Slit3 from M2 macrophages induces browning of WAT by increasing sympathetic nerve density and activity [16]. In our study, we observed that HIIT significantly increased the expression of Slit3 in the iWAT (Figure 6A). Furthermore, the expression levels of neurotrophins, such as brain-derived neurotrophic factor (BDNF) and nerve growth factor (NGF), were significantly higher in the iWAT of mice in the T2DM-EX group compared to non-exercised T2DM mice (Figure 6B,C).

To investigate the effect of exercise on local sympathetic nerve density and growth in iWAT, we examined sympathetic nerve density using immunofluorescence with an anti-tyrosine hydroxylase (TH) antibody, given that TH is a marker of sympathetic innervation and activation (Figure 6D). The results showed a significant increase in the number of TH-positive neurons in the iWAT of mice in the T2DM-EX group, accompanied by upregulation of TH protein and mRNA expression (Figure 6E–G). Additionally, we assessed the expression of growth-associated protein 43 (GAP43), a major marker involved in axon growth and nerve terminal development. Exercise significantly increased the levels of GAP43 mRNA (Figure 6H). These findings suggest that exercise promotes local sympathetic growth and increases sympathetic nerve density in iWAT, which may be associated with the release of Slit3 from M2 macrophages.

### 3.7. Linking Macrophage Phenotype to Local Sympathetic Nerves

**Figure 7:**
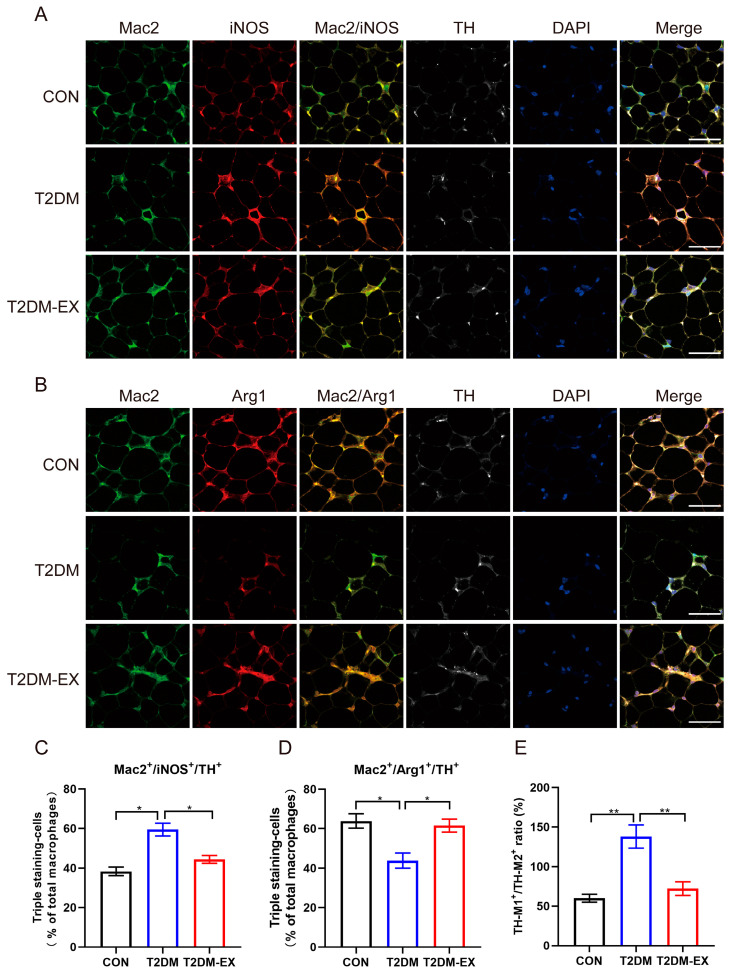
Exercise-induced aggregation of M2-like macrophages surrounding sympathetic nerves. Immunofluorescence staining of iWAT for TH (white), Mac2 (green), and iNOS (red, **A**) or Arg1 (red, **B**), and stained nuclei with DAPI (blue). Scale bar = 50 μm. (**C**–**E**) In pan-macrophages (Mac2^+^), the proportion of TH^+^Mac2^+^iNOS^+^ cells or TH^+^Mac2^+^Arg1^+^ cells and the ratio of the two. Three samples per group, 6 fields per section. * *p* < 0.05, ** *p* < 0.01. Data are expressed as mean ± SEM.

Recent evidence suggests that macrophages interact indirectly [16,17] or directly [18] with sympathetic nerves to regulate lipolysis and browning. In the present study, to explore the relationship between macrophage phenotype and sympathetic nerves, we stained iWAT with the sympathetic nerve marker TH and the pan-macrophage marker Mac2. Our data revealed that the TH signal almost completely overlaps the Mac2 signal in the area around adipocytes in the inguinal fat (Figure 7A,B), suggesting that macrophage is involved in sympathetic nerve function. By identifying specific macrophage phenotypic markers (iNOS and Arg1), we also assessed the relationship of macrophage subsets to TH (Figure 7A,B). Laser scanning confocal imaging revealed a significant increase in the proportion of M1-like macrophages (Mac2^+^iNOS^+^) co-localized with TH in iWAT pan-macrophages of T2DM mice (Figure 7C,E). In contrast, exercise induces more M2-like macrophages to accumulate near TH-positive sympathetic nerve endings (Figure 7D,E).

## 4. Discussion

In this study, we found that 8 weeks of HIIT induced browning of subcutaneous adipose tissue and improved glucose metabolism in T2DM mice. This result was associated with increased M2 macrophage and sympathetic nerve density in adipose tissue. Our results provide additional evidence that HIIT may be a feasible non-pharmacological therapeutic intervention to at least delay the progression of T2DM.

Exercise is widely recommended as a primary strategy for managing T2DM. Existing guidelines for metabolic diseases, including T2DM and obesity, typically suggest resistance training and a minimum of 150 min of moderate-intensity exercise at least twice a week. However, many patients struggle to allocate sufficient time for exercise and establish a regular habit [19]. Recent research has indicated that HIIT can deliver comparable or even superior health benefits to moderate-intensity exercise within a shorter duration. When compared to continuous walking with matched energy expenditure, HIIT training has shown improvements in fitness levels, body composition, and glycemic control [20]. Additionally, in adults with T2DM, HIIT has been linked to enhanced insulin sensitivity and pancreatic β-cell function [21]. Our study provided further evidence on the positive effects of HIIT in the context of T2DM. Our data demonstrated that HIIT reduced fasting glucose levels and improved glucose intolerance and insulin resistance in T2DM mice. Furthermore, HIIT led to significant reductions in body weight and body fat percentage. These findings align with previous research demonstrating that HIIT is associated with improvements in maximal oxygen uptake, insulin sensitivity, body weight, and body fat percentage [22].

Our results show that HIIT induces beige adipogenesis, leading to an increase in thermogenic activity in the iWAT of T2DM mice. The activation of beige adipocytes promotes glucose uptake, fatty acid oxidation, and lipolysis, thereby enhancing insulin sensitivity and reducing blood glucose, blood lipids, and fat mass [23]. This process plays a crucial role in preventing obesity and T2DM. Therefore, the browning of iWAT may serve as the primary mechanism linking HIIT training to the improvement of T2DM.

As the most abundant immune cells in adipose tissue, macrophages play an important role in various physiological functions, including inflammation and browning. Many studies have demonstrated that exercise exerts anti-inflammatory effects by inhibiting macrophage infiltration into adipose tissue and reducing the expression of associated chemokines [15,24,25,26,27]. However, the effects of acute swimming exercise on macrophage infiltration in the epididymal fat pad were found to be negligible [28], and endurance exercise was found to increase the number of CD68^+^ macrophages in the subcutaneous adipose tissue of obese individuals [29].

In our study, we observed that HIIT suppressed inflammation in the iWAT of T2DM mice while increasing the expression of chemokines MCP-1 and CXCL14. Notably, CXCL14 induces browning of white adipose tissue by attracting M2 macrophages and promoting their polarization [14]. We further examined the infiltration and polarization status of macrophages and found that HIIT induced increased expression of pan-macrophage markers (Mac2, F4/80, CD11b) while promoting the upregulation of M2 macrophage markers (Arg1, CD206) in iWAT. This result indicates that HIIT induces increased macrophage infiltration and promotes M2 macrophage polarization in the iWAT of T2DM mice, contributing to the improvement of the inflammatory state. It should be noted that the M1/M2 paradigm is often considered an oversimplified dichotomous division and should rather be considered as a continuum [30,31,32].

IL-25-induced polarization of M2 macrophages promoted sympathetic growth in subcutaneous fat [33]. M2 macrophages have been reported to stimulate local sympathetic activation and growth in iWAT by releasing Slit3 [16], and our results showed that HIIT significantly increased Slit3 gene expression. Voluntary exercise reduces the expression of sympathetic markers in mesenteric adipose tissue of rats with polycystic ovary syndrome [34], and aerobic exercise attenuates hyperexcitation of sympathetic nerves in perivascular adipose tissue of mice after transverse aortic constriction [35]. However, the effects of exercise on local sympathetic nerves in subcutaneous adipose tissue are unclear and less reported. We further examined the expression of neurotrophic factors and found that HIIT significantly increased the mRNA levels of BDNF and NGF. Myeloid-derived BDNF is critical for the maintenance of iWAT innervation [36], and NGF, although of unknown origin, is involved in the promotion of sympathetic nerve density [37]. The expressions of sympathetic marker TH and nerve growth marker gene GAP43 were significantly upregulated. These results show that HIIT significantly increased nerve density in the iWAT of T2DM mice, which may be associated with macrophages. In addition, our data show that sympathetic nerves are anatomically close to macrophages, and HIIT induces more M2 macrophages to co-localize with TH. Therefore, we can hypothesize that the M2 macrophage is a reasonable candidate for mediating HIIT-enhanced sympathetic nerve density and activity.

The study’s limitations include the absence of isolation of macrophages and sympathetic nerves from adipose tissue during the experiment. Additionally, the molecular mechanisms underlying exercise-induced macrophage polarization require further exploration. Another limitation is the lack of evaluation of protein levels in some parameters. While protein levels are predominantly determined by transcript concentrations under steady-state conditions, dynamic phases such as cellular differentiation or stress response may lead to deviations from this correlation. Therefore, future investigations should assess the protein levels of these cytokines using ELISA or immunoblotting for greater accuracy in the studies.

In conclusion, this study reveals that HIIT promotes adipose tissue browning and enhances glucose metabolism in T2DM mice. HIIT leads to improvements in fasting glucose, glucose tolerance, and insulin tolerance, along with reductions in body weight and fat mass. HIIT also induces M2 macrophage polarization, upregulates the expression of neurotrophic factors and Slit3, and promotes sympathetic nerve growth and density. Our findings highlight the potential of HIIT as a promising therapeutic strategy for T2DM by elucidating the underlying molecular mechanisms involved in adipose tissue browning and metabolic improvements.

## Figures and Tables

**Table 1:** Primers used for quantitative real-time PCR analysis.

Gene Name	Forward Primer Sequences	Reverse Primer Sequences
Cidea	5′-GCCTGCAGGAACTTATCAGC-3′	5′-AGAACTCCTCTGTGTCCACCA-3′
COX8B	5′-TGTGGGGATCTCAGCCATAGT-3′	5′-AGTGGGCTAAGACCCATCCTG-3′
NRF-1	5′-GCCGTCGGAGCACTTACT-3′	5′-CTGTTCCAATGTCACCACC-3′
PGC1α	5′-GAAAGGGCCAAACAGAGAGA-3′	5′-GTAAATCACACGGCGCTCTT-3′
COX4	5′-TGAATGGAAGACAGTTGTGGG-3′	5′-GATCGAAAGTATGAGGGATGGG-3′
TMEM26	5′-ACCCTGTCATCCCACAGAG-3′	5′-TGTTTGGTGGAGTCCTAAGGTC-3′
PRDM16	5′-CCTAAGGTGTGCCCAGCA-3′	5′-CACCTTCCGCTTTTCTACCC-3′
TNFα	5′-GACGTGGAACTGGCAGAAGAG-3′	5′-TTGGTGGTTTGTGAGTGTGAG-3′
IL-10	5′-GCTCTTACTGACTGGCATGAG-3′	5′-CGCAGCTCTAGGAGCATGTG-3′
MCP-1	5′-TTAAAAACCTGGATCGGAACCAA-3′	5′-GCATTAGCTTCAGATTTACGGGT-3′
Mac2	5′-AGACAGCTTTTCGCTTAACGA-3′	5′-GGGTAGGCACTAGGAGGAGC-3′
F4/80	5′-TTGTACGTGCAACTCAGGACT-3′	5′-GATCCCAGAGTGTTGATGCAA-3′
CD11b	5′-TAATGACTCTGCGTTTGCCCTG-3′	5′-ATTGGAGCTGCCCACAATGAG-3′
iNOS	5′-GTTCTCAGCCCAACAATACAAGA-3′	5′-GTGGACGGGTCGATGTCAC-3′
CD11c	5′-CTGGATAGCCTTTCTTCTGCTG-3′	5′-GCACACTGTGTCCGAACTC-3′
Arg1	5′-CTCCAAGCCAAAGTCCTTAGAG-3′	5′-AGGAGCTGTCATTAGGGACATC-3′
CD206	5′-CTCTGTTCAGCTATTGGACGC-3′	5′-CGGAATTTCTGGGATTCAGCTTC-3′
Slit3	5′-GCGCGATTTGGAGATCCTCA-3′	5′-TGGAGTGTAGACGCAGAGTCC-3′
BDNF	5′-TCATACTTCGGTTGCATGAAGG-3′	5′-AGACCTCTCGAACCTGCCC-3′
GAP43	5′-TGGTGTCAAGCCGGAAGATAA-3′	5′-GCTGGTGCATCACCCTTCT-3′
TH	5′-GTCTCAGAGCAGGATACCAAGC-3′	5′-CTCTCCTCGAATACCACAGCC-3′
GAPDH	5′-AACTTTGGCATTGTGGAAGG-3′	5′-ACACATTGGGGGTAGGAACA-3′

## Data Availability

The data that support the findings of this study are available from the corresponding author upon reasonable request due to privacy or ethical restrictions.

## Supplementary Materials

The following supporting information can be downloaded at: https://www.mdpi.com/article/10.3390/biom14030246/s1, Figure S1: Original images of Figure 6F.
