# Supplementary figures and images for: HIIT Promotes M2 Macrophage Polarization and Sympathetic Nerve Density to Induce Adipose Tissue Browning in T2DM Mice
Source: Biomolecules. 2024 Feb 20;14(3):246. doi: 10.3390/biom14030246 (PMC10968334; doi:10.3390/biom14030246)

**Figure S1:**

The original image of Figure 6F:

TH: 60KD

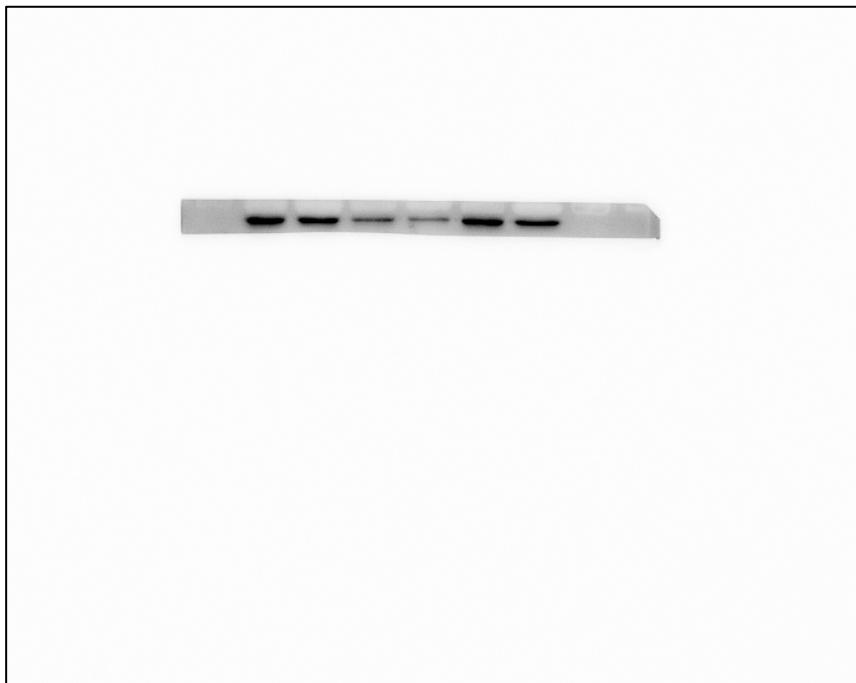

$\beta$ -actin: 42KD

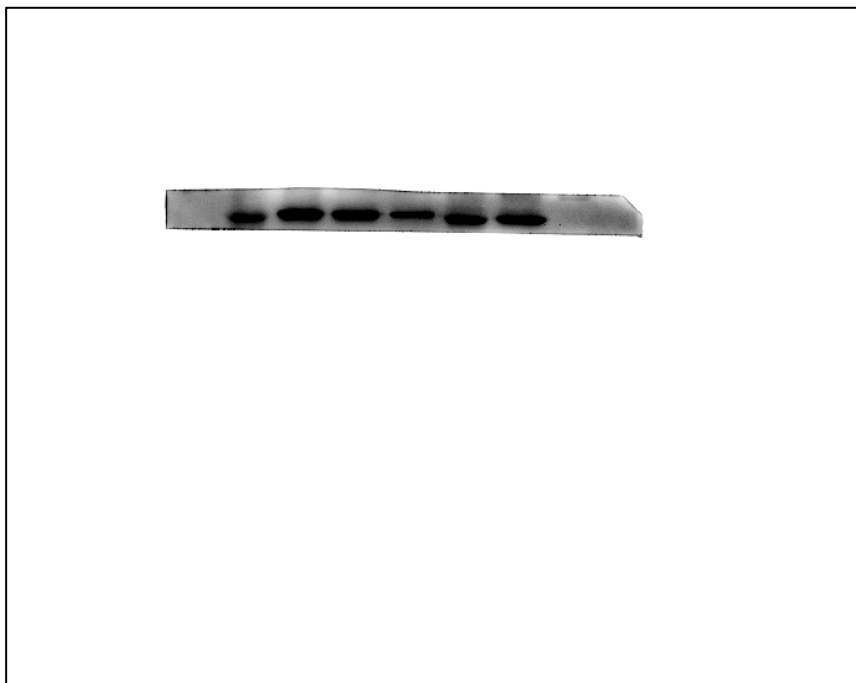

Supplement: Supplementary file 1 [file biomolecules-14-00246-s001.zip › biomolecules-2860156-supplementary.pdf]
